# Whole-genome sequencing, annotation, and biological characterization of a novel Siphoviridae phage against multi-drug resistant *Propionibacterium acne*

**DOI:** 10.3389/fmicb.2022.1065386

**Published:** 2023-01-04

**Authors:** Danxi Liao, Jian Zhang, Ruolan Liu, Kui Chen, Yuanyuan Liu, Yuming Shao, Xi Shi, Yiming Zhang, Zichen Yang

**Affiliations:** ^1^Department of Plastic and Cosmetic Surgery, Xinqiao Hospital, The Second Affiliated Hospital, Army Medical University (The Third Military Medical University), Chongqing, China; ^2^Department of Clinical Laboratory, Xinqiao Hospital, The Second Affiliated Hospital, Army Medical University (The Third Military Medical University), Chongqing, China; ^3^Cadet Brigade 4, College of Basic Medicine, Army Medical University (The Third Military Medical University), Chongqing, China; ^4^Department of Microbiology, College of Basic Medicine, Army Medical University (The Third Military Medical University), Chongqing, China

**Keywords:** *Propionibacterium acne*, bacteriophage, severe acne vulgaris, antibiotics-resistant, phage therapy

## Abstract

Antibiotics-resistant *Propionibacterium acne* (*P. acne*) causes severe acne vulgaris, serious public health, and psychological threat. A new lytic bacteriophage (phage), φPaP11-13, infecting *P. acne,* was isolated from the sewage management center of Xinqiao Hospital. It can form transparent plaque with diameters of 1.0 ~ 5.0 mm on the double-layer agar plate, indicating a robust lytic ability against its host. Transmission electron microscopy (TEM) showed that φPaP11-13 belonged to the Siphoviridae family (head diameter 60 ± 4.5 nm, tail length 170 ± 6.4 nm, tail width 14 ± 2.4 nm). The one-step growth curve showed the incubation period was 5 h, and the burst size was 26 PFU (plaque-forming unit)/cell. Moreover, it exhibited tolerance over a broad range of pH and temperature ranges but was utterly inactivated by ultraviolet (UV) irradiation for 1 h. The whole-genome sequencing results revealed φPaP11-13 had a linear dsDNA with 29,648 bp length. The G/C content was 54.08%. Non-coding RNA genes and virulence factors were not found. Forty five open reading frames (ORFs) were identified after online annotation. This study reports a novel *P. acne* phage φPaP11-13, which has a robust lytic ability, no virulence factors, and good stability. The characterization and genomic analysis of φPaP11-13 will develop our understanding of phage biology and diversity and provide a potential arsenal for controlling antibiotics-resistant *P. acne*-induced severe acne vulgaris.

## Introduction

Acne vulgaris is the eighth most prevalent disease worldwide, and 85% of the patients are young people aged 12–24 (Tan and Bhate, 2015; Lynn et al., 2016). Severe acne vulgaris mainly occurs in the frontal region, chest, back, and shoulder, where people always experience horrible feelings (Choi et al., 2012). Although acne vulgaris is not a fatal disease, the disfiguring and recurrent consequence brings psychosocial problems, particularly in severe cases (Silverberg and Silverberg, 2014). Not to mention that improper treatment aggravates lesions and irritates immunological reactions (Lavers and Courtenay, 2011).

*Propionibacterium acnes* (*P. acnes*), also known as *Cutibacterium acnes,* infection or imbalance of this commensal bacterium is one of the critical causes of the aggravation of acne vulgaris (Dréno et al., 2018). Moreover, severe acne induced by antibiotic-resistant *P.acne* infection is more difficult to treat (Nakase et al., 2017). According to *Treatment Guidelines from the AAD*, the first-line treatment for severe acne vulgaris caused by *P. acne* includes oral isotretinoin and tetracyclines (Hauk, 2017). However, tetracycline requires a long period of application (often > 6 months) (Fox et al., 2016), which inevitably arouses side effects on the body (Nakase et al., 2018). Abundant studies have shown that tetracycline resistance in *P. acne* is becoming increasingly evident (Nakase et al., 2017). Moreover, the long therapy duration increases the likelihood of overgrowth of antibiotic resistance in *P. acne* (Oprica et al., 2004). Mendoza even reported tetracycline-resistant *P. acne* isolated from the faces of patients who had never taken tetracycline (Mendoza et al., 2013). Thus, there is an urgent need for alternative therapies against antibiotics-resistant *P. acne*-induced severe acne vulgaris.

Phages are viruses that infect, parasitize, and lyse bacteria with precision and efficiency (Gordillo Altamirano and Barr, 2019). Since the 1920s, phages with high bactericidal activity have been used to treat human bacterial infectious diseases (Kortright et al., 2019). In recent years, antimicrobial resistance (AMR) has become a growing problem, and this “phage therapy” has received renewed attention (Kortright et al., 2019). *P. acne* phage has a long history. The *P. acne* phage was first identified in 1964 (Brzin, 1964). Subsequently, Zierdt et al. (1968) conducted in-depth studies and found it had similar morphology, broad host range, and good stability (Zierdt, 1974; Farrar et al., 2007). Phages can treat bacterial infections regardless of antibiotics-resistance (Yin et al., 2017; Yang et al., 2019a). There were reports on *P. acne* phage effectively treating multi-drug-resistant *P. acne*-induced acne vulgaris in animal experiments (Lam et al., 2021) and clinical trials (Golembo et al., 2022), indicating potential clinical values. However, phage therapy for treating severe acne vulgaris induced by antibiotic-resistant *P. acne* is insufficient. More research is needed on the phage genomic backgrounds and future host-phage interactions.

We isolated and identified a novel *P. acne* phage with solid lytic abilities against a clinical antibiotics-resistant *P. acne* strain Pacne11-13. The biological characterization and genome annotation may provide vital information for the application of antibiotics-resistant *P. acne*-induced severe acne vulgaris treatment and further studies.

## Materials and methods

### Bacterial gene sequencing and antimicrobial susceptibility test

The clinical *P. acne* strain Pacne11-13 was separated from facial abscesses in patients with severe acne vulgaris, identified by VITEK 2 Compact system (BioMérieux, France) (Dréno et al., 2018). The gene sequencing of Pacne11-13 was performed using PacBio Sequel and Illumina NovaSeq PE150 platforms. Software Diamond and the Antibiotic Resistance Genes Database (ARDB) (Liu and Pop, 2009) combined the sequenced genes with the antibiotic-resistance functional annotation information to obtain the results. The antibiotics susceptibility test of Pacne11-13 (tetracycline, aminoglycosides, cephalosporins, and clindamycin) was tested using the disk diffusion method (Rosco Neo-Sensitab) (Schuetz, 2014). The Pacne11-13 was grown in a BHI medium (Brain-Heart-Infusion, Oxoid, United Kingdom) and incubated at 37°C in a hypoxic incubator (HR900-IIB2, ESCO).

### Phage isolation and purification

*P. acne* phage was screened and enriched as previously described (Huang et al., 2014; Yang et al., 2019b), with some alterations. Briefly, sewage water samples from the sewage management center at Xinqiao Hospital used as sources of phages were collected and then centrifuged (Ultracentrifuge AG22331, Eppendorf, German) at 10000 × *g* for 15 min at 4°C. The supernatant was filtered through 0.22-μm PES needle filters (Millipore, United States) to remove any remaining bacteria. Then *P. acne* was cultured to the exponential phase (OD600 = 0.6) and was taken 1 ml to mix with 10 ml sewage filter liquor. The growth curve of host bacteria is in Supplementary Figure S1. Then the mixture was added to a 100 ml BHI liquid medium and co-cultivated for 24 h on a hypoxic rocking platform at 37°C to enrich any *P. acne* phages in the sewage samples. The next day, this mixture was centrifuged at 10,000 × *g* for 10 min at 4°C, and the supernatant was passed through 0.22-μm PES needle filters. The phage was purified and separated by picking single-plaque with the double-layer agar method (Shi et al., 2021). And the resulting isolation was stored at 4°C.

### Transmission electron microscopy

Three 3-μL aliquots of the high-titer phage preparations (10^10^ ~ 10^13^ PFU /mL) were pipetted on carbon-coated copper grids as instructed (Shi et al., 2021). After allowing the phages to adsorb for 1 min, the grids were stained with 2% uranyl acetate (pH 4.2) for 30 s. Then the morphology of φPaP11-13 was observed and photographed under TEM (Hitachi HTT700, Japan). The heads and tails of 5 individual phage particles were measured by Image-Pro Plus6.0 image analysis software (Media Cybernetics 6.0, United States) to calculate the averages and standard errors for the dimensions (Yang et al., 2019a).

### The optimal MOI and optimal adsorption time

The multiplicity of infection (MOI) refers to the ratio of the plaque-forming unit (PFU) to the colony-forming unit (CFU). Firstly, the titer of *P. acne* was adjusted to 1 × 10^8^ CFU/ml and φPaP11-13 to 1 × 10^8^ PFU/mL. Then mixed the *P. acne* with the φPaP11-13 in proportion (10.000, 1.0000, 0.1000, 0.0100, 0.0010, and 0.0001). After 24 h, phage titers were measured by the spot test (Shi et al., 2020). The proportion with the highest phage titer was the optimal MOI.

The phages and bacteria were mixed at the optimal MOI tested in the above experiments. Placed it in a hypoxia incubator and incubated for 5, 10, and 15 min. It was the optimal adsorption time when the phage titer was the lowest. Those experiments were repeated three times, and three parallel averages were taken under per-scale titers. The measurement data conformed to the normal distribution and were expressed as mean ± standard errors.

### One–step growth curve

A one-step growth curve, including incubation, lysis, and platform period, can demonstrate the phage life cycle. φPaP11-13 was mixed with *P. acne* at optimal MOI (0.0100) and adsorption at 37°C for optimal adsorption time (10 min). The mixture was centrifuged at 10000 × *g* for 60 s to clear unabsorbed phages from the supernatant. Then the residue was washed twice with BHI liquid medium on ice, resuspended by 5 ml BHI liquid medium, and cultured in a hypoxic incubator at 37°C. Samples were taken out at 0, 1, 2, 3, 4, 5, 6, 7, 8, 9, 10, 11, and 12 h after injection, and the spot test measured the titer. According to previous reports, the burst size was calculated based on the one-step growth curve (Kropinski, 2018). This experiment was performed in triplicate.

### Stability tests of φPaP11-13

To test the stability of φPaP11-13 under various conditions, we measured its titer at different temperatures, pH, UV, and Chloroform conditions, using our previously described method with some alterations (Shi et al., 2020). The φPaP11-13 solution was incubated in a water bath at different temperatures (−20, 4, 37, 50, 60, 70, and 80°C), and phage titers were measured after 1 h. Likewise, to measure the pH stability of φPaP11-13, samples were incubated at different pH values (1, 2, 3, 4, 5, 6, 7, 8, 9, 10, 11, 12, 13) at 37°C and samples were taken out for phage titer determination after 1 h. Then, to measure the UV stability of the phage. The phage sample was added to a 16-well plate, and the cover was removed and placed under UV light. After 0, 5, 10, 30, and 60 min of irradiation, the phage titers were measured. Finally, measured the stability of Chloroform. The spot test was used to measure the phage titer in the above experiments, and those experiments were repeated three times. The data were analyzed by GraphPad Prism version 9 for Windows (GraphPad Software, La Jolla, CA, United States).

### Host range

Spot test and double-layer agar plate were used to determine the host range of φPaP11-13. Sixteen strains of *P. acnes* isolated from facial abscesses in patients were used for the host range experiment. 300 μl host bacteria with logarithmic phase (OD600 = 0.6) of each strain was mixed with 5 ml semi-solid agar and then poured into the bottom agar plate. 5 μl φPaP11-13 solution (10^10^ PFU/ml) was dropped onto the upper agar with more than three spots per plate. After the phage suspension was adsorbed, the plate was incubated in a hypoxic incubator at 37°C for 24 h.

### DNA extraction and genome sequencing

Phage DNA was extracted using the standard Protease-K/SDS DNA extraction method (Shi et al., 2020). The nucleic acid concentration was measured using a NanoDrop spectrophotometer (ND-1000, Wilmington, DE, United States). This genome sequencing adopted the Whole Genome Shotgun (WGS) strategy to construct libraries of different insertions and utilized Next-Generation Sequencing (Dréno et al.) technology based on the Illumina NovaSeq Sequencing platform. Paired-end (PE) sequencing was performed on these libraries.

### Whole genome sequence annotation and analysis

Phageterm (Garneau et al., 2017) was used to predict the genome ends of φPaP11-13, and GeneMarkS (Brettin et al., 2015), RAST (Aziz et al., 2008), and PHASTER (Arndt et al., 2016) were used as genome prediction software to predict ORFs of the assembled φPaP11-13 genome sequence. Then the predicted ORF was proofread using the Basic Local Alignment Search Tool (BLAST) of NCBI.^1^ The annotated nucleotide sequence of the φPaP11-13 genome was submitted to the NCBI database under the accession number: ON557706, and the CGView Server BETA^2^ (Stothard et al., 2019) was used to visualize the φPaP11-13 genome. To analyze the evolution trend and relation of φPaP11-13 with other known phages, Mauve 20,150,226 was used for collinearity analysis (Darling et al., 2004), and Mega-X 10. 0. 2 was used for phylogenetic tree analysis of the major terminal large subunit sequences (Kumar et al., 2018).

### Identification of phage proteins

Liquid nitrogen was added to the phage particle, and then it was ground to powder. Then the phage particle was lysed by ultrasound, and a BCA kit measured its protein concentration. 5 μl of 4 × loading buffer and 2% SDS were added to the sample and then electrophoretic. The silver dyeing method was transferred into the chromogenic solution for about 10 min at room temperature, and clear bands were finally visible. Then the cut protein bands were tested by LC–MS/MS system (UPLC, EASY-nLC 1,200 system, PTM BioLab, Hangzhou) for phage major protein analysis. Then cross-checking underwent between LC–MS/MS results and whole genome annotation.

## Results

### Antimicrobial resistance genes annotation and AST analysis of clinically isolated strain Pacne11-13

The whole genome of Pacne11-13 underwent sequencing. And AMR genes were annotated and listed in Table 1. The bacteria carried 17 AMR genes, including drug efflux-related, antibiotic target protection, antibiotic inactivation, and antibiotic target replacement genes, resulting in resistance against tetracycline, aminoglycosides, fluoroquinolones, macrolides, rifamycin, peptide antibiotics and cephalosporin, and other antibiotics. In addition, the AST results indicated that Pacne11-13 was multi-drug resistant to tetracycline, aminoglycosides, cephalosporins, and clindamycin (Table 2).

**Table 1:** Annotation of bacterial antibiotic-resistance genes of Pacne11-13.

Gene ID	ARO name	AMR gene family	Drug class	Resistance mechanism
GM000034	mdtN	Major facilitator superfamily (MFS) antibiotic efflux pump	Acridine dye; disinfecting agents and intercalating dyes; nucleoside antibiotic	Antibiotic efflux
GM000140	kdpE	kdpDE	Aminoglycoside antibiotic	Antibiotic efflux
GM000202	efrB	ATP-binding cassette (ABC) antibiotic efflux pump	Fluoroquinolone antibiotic; macrolide antibiotic; rifamycin antibiotic	Antibiotic efflux
GM000203	efrA	ATP-binding cassette (ABC) antibiotic efflux pump	Fluoroquinolone antibiotic; macrolide antibiotic; rifamycin antibiotic	Antibiotic efflux
GM000393	mtrA	Resistance-nodulation-cell division (RND) antibiotic efflux pump	Macrolide antibiotic; penam	Antibiotic efflux
GM000404	adeH	Resistance-nodulation-cell division (RND) antibiotic efflux pump	Fluoroquinolone antibiotic; tetracycline antibiotic	Antibiotic efflux
GM000492	AAC (3)-IIb	AAC (3)	Aminoglycoside antibiotic	Antibiotic inactivation
GM000640	ugd	Pmr phosphoethanolamine transferase	Peptide antibiotic	Antibiotic target alteration
GM000860	MexD	Resistance-nodulation-cell division (RND) antibiotic efflux pump	Aminocoumarin antibiotic; aminoglycoside antibiotic; cephalosporin; diaminopyrimidine antibiotic; fluoroquinolone antibiotic; macrolide antibiotic; penam; phenicol antibiotic; tetracycline antibiotic	Antibiotic efflux
GM001163	Escherichia coli ampC1 beta-lactamase	AmpC-type beta-lactamase	Cephalosporin; penam	Antibiotic inactivation
GM001249	RbpA	RbpA bacterial RNA polymerase-binding protein	Rifamycin antibiotic	Antibiotic target protection
GM001372	mtrA	Resistance-nodulation-cell division (RND) antibiotic efflux pump	Macrolide antibiotic; penam	Antibiotic efflux
GM001687	Trimethoprim-resistant dihydrofolate reductase DfrA43	Trimethoprim resistant dihydrofolate reductase dfr	Diaminopyrimidine antibiotic	Antibiotic target replacement
GM001792	AAC (3)-IIb	AAC (3)	Aminoglycoside antibiotic	Antibiotic inactivation
GM001899	Bifidobacterium adolescentis rpoB mutants conferring resistance to rifampicin	Rifamycin-resistant beta-subunit of RNA polymerase (rpoB)	Peptide antibiotic; rifamycin antibiotic	Antibiotic target alteration; antibiotic target replacement
GM002057	arlR	Major facilitator superfamily (MFS) antibiotic efflux pump	Acridine dye; disinfecting agents and intercalating dyes; fluoroquinolone antibiotic	Antibiotic efflux
GM002320	msbA	ATP-binding cassette (ABC) antibiotic efflux pump	Nitroimidazole antibiotic	Antibiotic efflux

ARO, antibiotic resistance ontology; AMR, antimicrobial resistance.

**Table 2:** Antimicrobial susceptibility test.

Antibiotics	Diameter of zone of inhibition (mm)
Tetracycline	6.2 + 2.4
Aminoglycosides	4.5–0.3
Cephalosporins	3.4 + 0.9
Clindamycin	2.9 + 0.2

Criteria: diameter of inhibition zone > 20 mm, high sensitivity; 10 ~ 20 mm, moderate sensitivity; < 10 mm, resistant.

### Plaque and morphology of φPaP11-13

**Figure 1:**
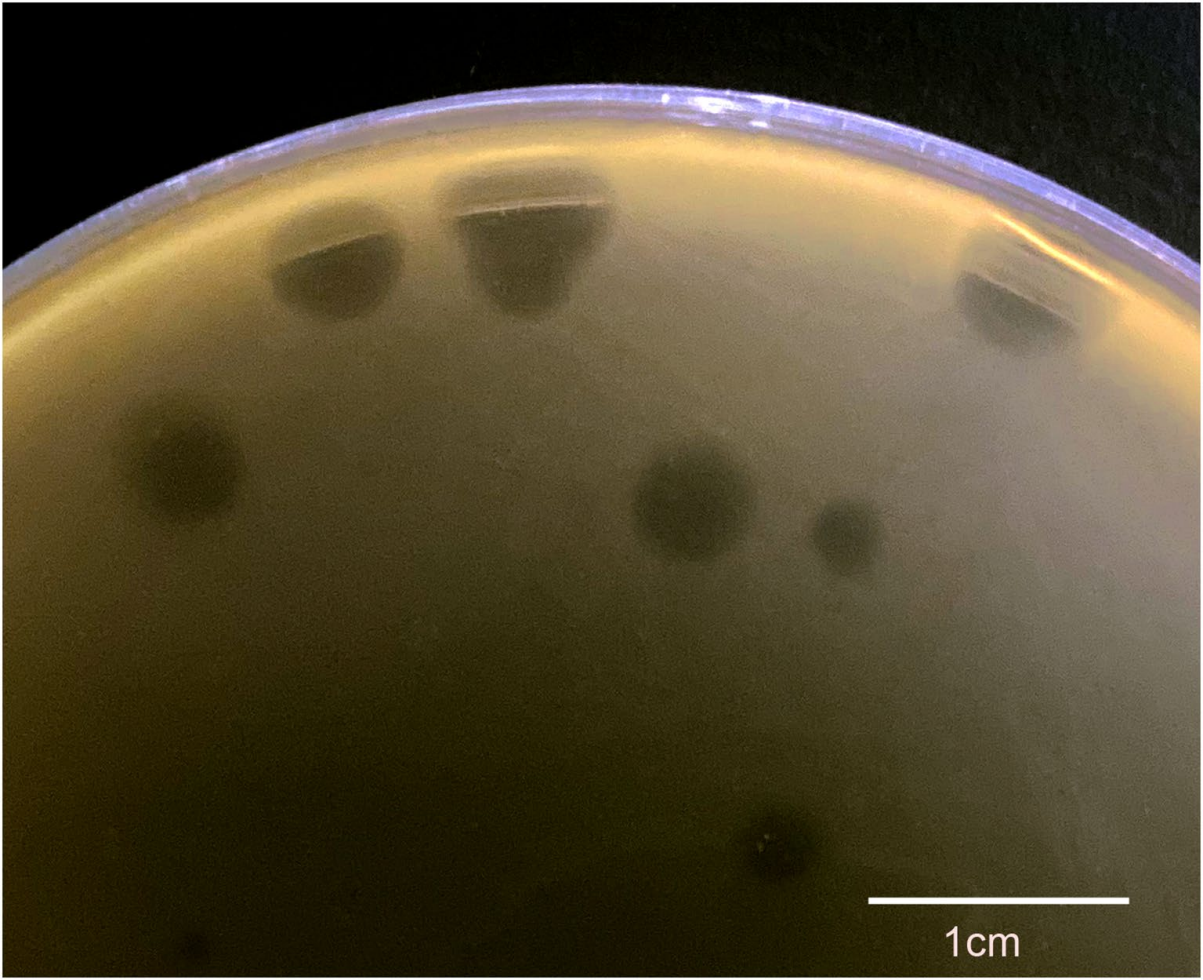
Plaque pattern formed by φPaP11-13 lysis *P. acne*. The scale bar is 1 cm.

**Figure 2:**
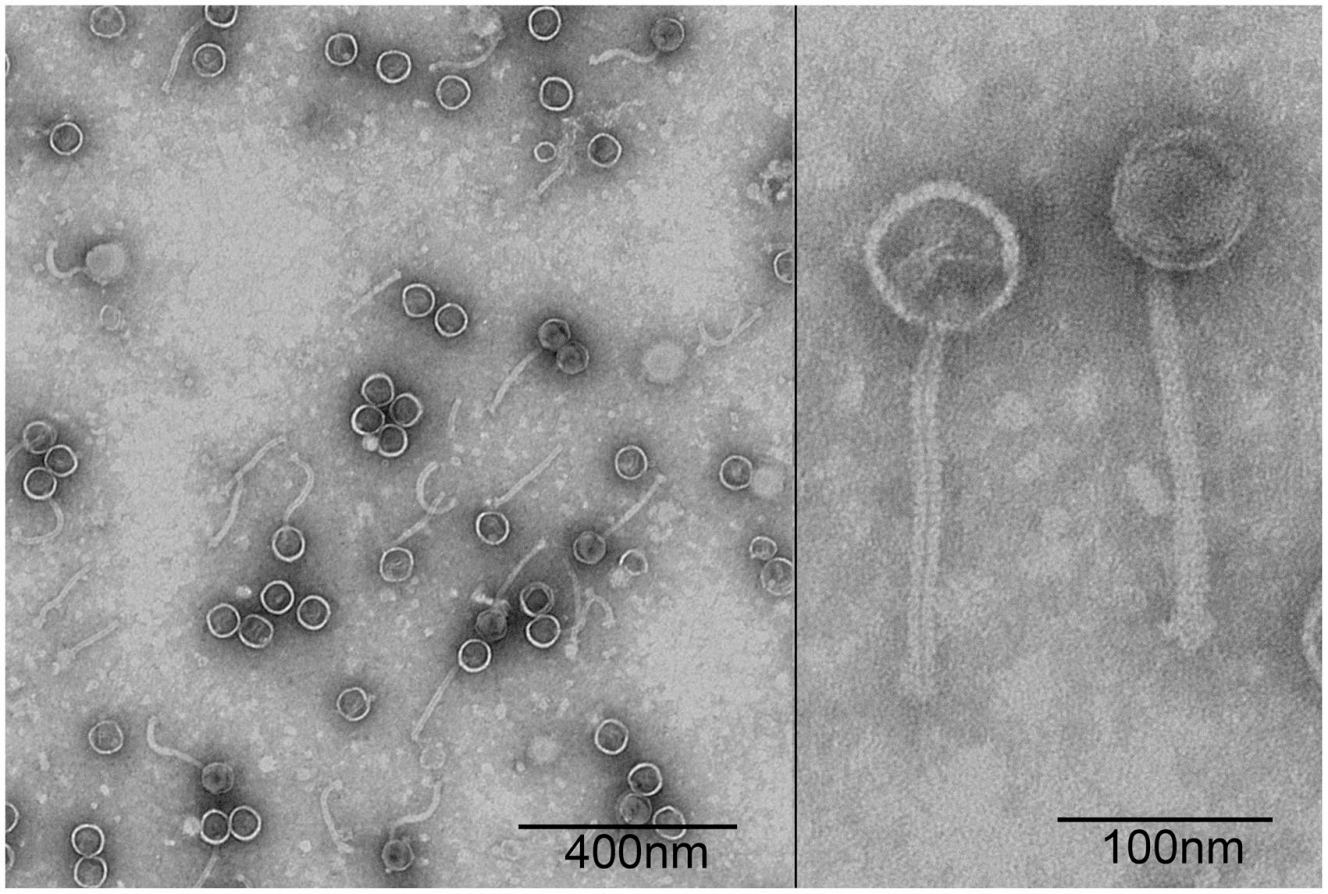
Transmission electron micrograph of φPaP11-13, negatively stained with 2% uranyl acetate.

A *P. acne* phage was isolated from the sewage management center at Xinqiao Hospital and named “φPaP11-13.” This phage can form transparent circular plaques (diameter ranges from 1.0 to 5.0 mm, Figure 1). TEM showed that φPaP11-13 had a polyhedron head (diameter 60 ± 4.5 nm) and an untraceable flexible tail (length 170 ± 6.4 nm, and width 14 ± 2.4 nm, Figure 2). These morphological features suggested that φPaP11-13 was a member of the Siphoviridae family.

### Optimal MOI and optimal adsorption time

**Figure 3:**
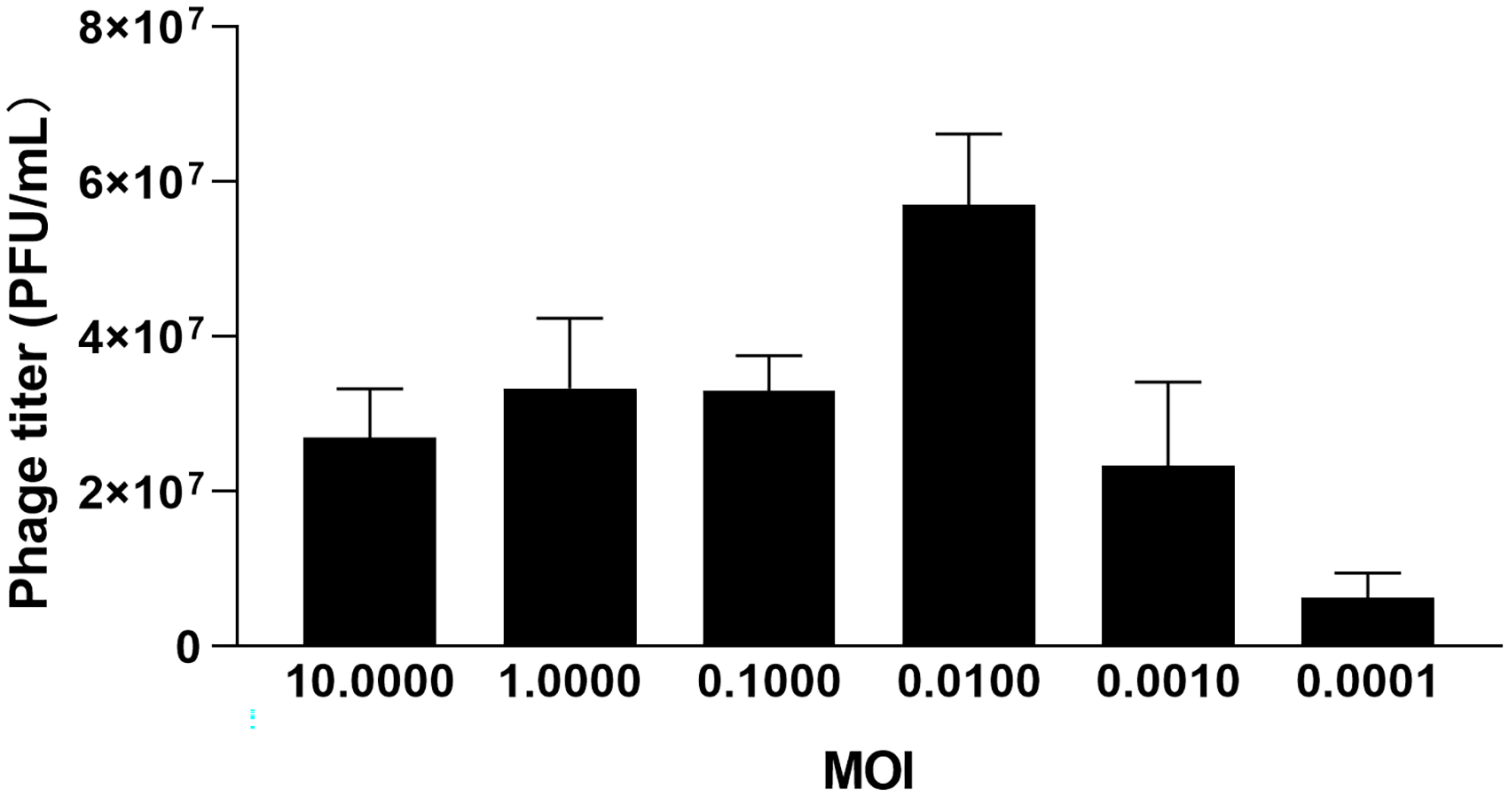
The MOI test of φPaP11-13.

Three groups of parallel experiments were conducted for MOI tests, and the optimal MOI of φPaP11-13 was 0.0100 (Figure 3). After 6 h of incubation, the titers of φPaP11-13 were 2.7 ± 0.6, 3.3 ± 0.9, 3.3 ± 0.4, 5.7 ± 0.9, 2.3 ± 1.1, 0.63 ± 0.3 × 10^7^ PFU/mL when the MOI was 10.0000, 1.0000, 0.1000, 0.0100, 0.0010, 0.0001. When incubated with host bacteria for 5, 10, and 15 min, the titers of φPaP11-13 respectively, were 20.0 ± 1.0, 1.56 ± 3.1, and 3.4 ± 0.9 × 10^7^ PFU/mL, suggesting an optimal adsorption time of φPaP11-13 at 10 min.

### One–step growth curve

**Figure 4:**
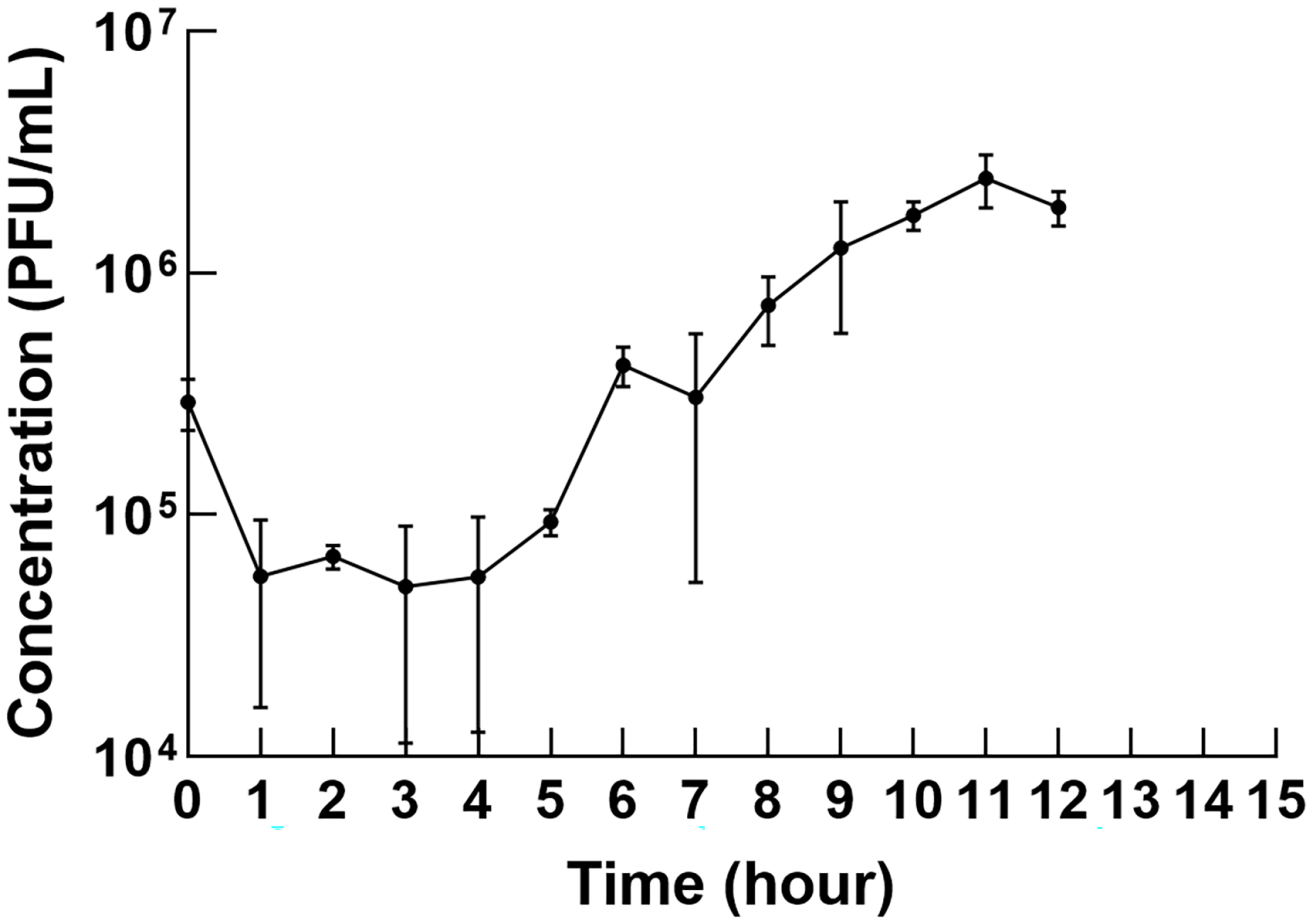
One-step growth curve. The incubation period was 5 h, and the burst size was 26 PFU/cell.

The one-step growth curve revealed the latency time and burst size of φPaP11-13. As shown in Figure 4, the titer decreased slightly in the first 10 min but then remained stable for the first 5 h but increased to a large degree in the next 5 h, indicating that phage was released by the infected cells. Thus, the change in phage titer showed that φPaP11-13 had a long incubation period of approximately 5 h, followed by a burst period, and then we can calculate the burst size. The phage titer reached a plateau about 10–12 h after co-culture. The burst size of φPaP11-13 is 26 PFU/cell.

### Stability of φPaP11-13

**Figure 5:**
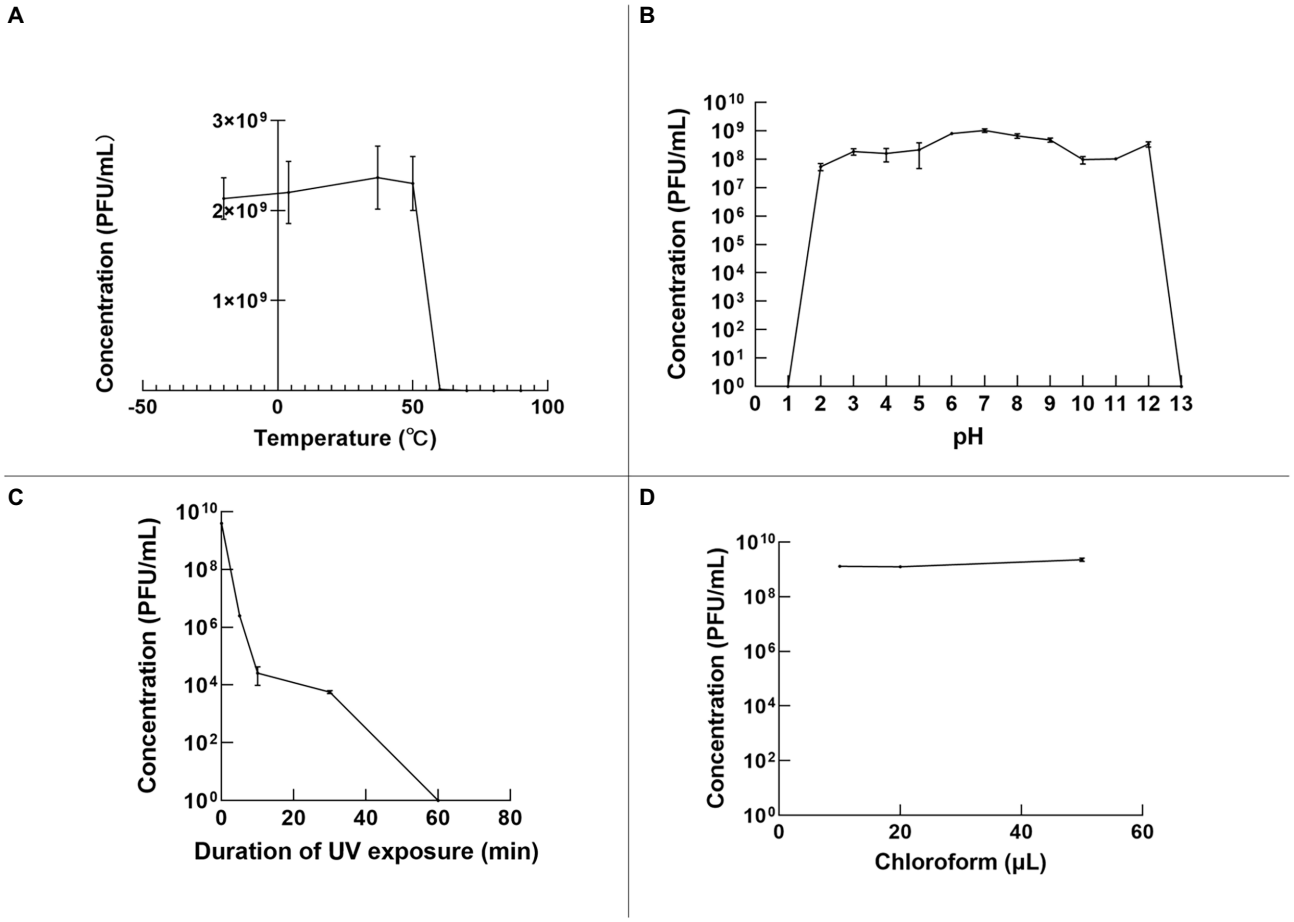
The stability of φPaP11-13, analyzed and plotted using GraphPad Prism version 9 **(A)** temperature stability. **(B)** pH stability. **(C)** Ultraviolet stability. **(D)** Chloroform stability.

The stability of φPaP11-13 was tested under various temperatures, pH, and UV irradiation. Temperature stability results showed that the phage titer remained still from −20 to 50°C. However, a temperature over 50°C would decrease phage titer rapidly to 0 (Figure 5A). In the acid–base stability test, the pH tolerance of φPaP11-13 was stable from 2.0 to 12.0 (Figure 5B). Moreover, the titer of φPaP11-13 decreased rapidly under UV irradiation and was utterly inactivated when the irradiation time reached 60 min (Figure 5C). Chloroform had no significant effect on phage activity (Figure 5D).

### Host range analysis

A total of 16 strains of *P. acne* were involved in the analysis of the lytic range of φPaP11-13. The results showed that various-sized transparent plaques could be seen on most double-layer agar plates. The lysed rate of φPaP11-13 was 100% (16/16), demonstrating the phage has a broad host range (Table 3).

**Table 3:** The host range of φPaP11-13.

*Propionibacterium acnes*	Lytic ability	Address
Pacne11-07	+	Xinqiao hospital
Pacne11-11	+	Xinqiao hospital
Pacne11-12	+	Xinqiao hospital
Pacne11-13	+	Xinqiao hospital
Pacne11-14	+	Xinqiao hospital
Pacne11-15	+	Xinqiao hospital
Pacne11-16	+	Xinqiao hospital
Pacne11-17	+	Xinqiao hospital
Pacne11-18	+	Xinqiao hospital
Pacne11-19	+	Xinqiao hospital
Pacne11-20	+	Xinqiao hospital
Cacne11-10	+	Xinqiao hospital
Cacne11-11	+	Xinqiao hospital
Cacne11-22	+	Xinqiao hospital
Cacne11-23	+	Xinqiao hospital
Cacne11-24	+	Xinqiao hospital

### General genome characteristics and ORF analysis

The linear dsDNA genome of φPaP11-13 consisted of 29,648 bp, with a G/C content of 54.08%. Forty five ORFs were predicted, accounting for 83.83% of the genome length, and the average length of each ORF was 606.22 bp. Among the 45 predicted ORFs and 20 ORFs were largely matched to genes with annotation functions in the GenBank database. No non-coding RNA, antibiotic resistance, or virulence factors were found in the phage genome (Table 4).

**Table 4:** Gene annotation results of φPaP11-13.

ORF	Direction	Start site	Stop site	Protein	Covery	*E*-value	Identity	Number
1	+	97	386	HNH endonuclease	99%	9.00*E*-53	85.42%	DAT38686.1
2	+	2,288	2,449	Hypothetical protein	98%	2.00*E*-20	75.44%	QPB11535.1
3	+	2,606	2,914	Hypothetical protein	99%	5.00*E*-64	95.10%	QPB11842.1
4	+	2,946	3,257	Hypothetical protein	99%	3.00*E*-67	95.15%	ATN87142.1
5	+	3,386	3,670	Hypothetical protein	98%	1.00*E*-57	89.36%	EAD6310181.1
6	+	3,754	4,290	Hypothetical protein	97%	3.00*E*-45	73.14%	ATN87138.1
7	+	4,315	4,542	Hypothetical protein	97%	5.00*E*-42	91.89%	DAQ10868.1
8	−	4,577	4,870	Hypothetical protein	98%	7.00*E*-13	84.54%	DAU50408.1
9	+	4,851	5,210	Hypothetical protein	99%	5.00*E*-64	94.96%	ATN90932.1
10	+	5,210	6,151	PD-(D/E)XK nuclease family protein	99%	0	90.42%	YP_009150041.1
11	+	6,148	6,558	Hypothetical protein	99%	2.00*E*-80	90.44%	ATN90930.1
12	+	6,610	7,068	Hypothetical protein	99%	5.00*E*-103	95.39%	YP_009152440.1
13	+	7,111	7,974	Hypothetical protein	94%	0	97.21%	YP_009152439.1
14	+	7,971	8,330	Hypothetical protein	99%	2.00*E*-77	94.96%	YP_008531712.1
15	+	8,474	9,199	DNA primase	99%	2.00*E*-165	96.27%	ASJ79909.1
16	+	9,244	9,810	Hypothetical protein	99%	5.00*E*-124	96.28%	ASJ79910.1
17	+	9,807	10,367	Hypothetical protein	99%	1.00*E*-128	93.01%	YP_009278006.1
18	+	10,383	10,545	Hypothetical protein	98%	5.00*E*-36	95.31%	QHB36816.1
19	+	10,542	11,588	Exonuclease	99%	0	95.98%	YP_009159989.1
20	+	11,598	11,918	Helix-turn-helix DNA binding domain protein	99%	6.00*E*-72	100.00%	ATN90304.1
21	+	11,930	12,217	Hypothetical protein	60%	5.00*E*-21	94.83%	YP_008531659.1
22	+	12,221	12,619	Sigma factor	98%	1.00*E*-90	96.97%	YP_008531794.1
23	+	12,624	12,899	Hypothetical protein	98%	8.00*E*-52	91.21%	DAS49464.1
24	−	13,022	13,384	Holin	99%	6.00*E*-75	93.33%	YP_009160209.1
25	−	13,391	14,254	Amidase	99%	0	96.17%	AGI12651.1
26	−	14,294	15,091	Collagen triple helix repeat protein	95%	2.00*E*-98	90.12%	DAU50379.1
27	−	15,094	15,357	Minor tail protein	98%	3.00*E*-46	93.10%	YP_009160161.1
28	−	15,403	16,221	H-type lectin domain-containing protein	99%	0	96.32%	YP_008531652.1
29	−	16,238	17,395	Hypothetical protein	99%	0	97.14%	DAS61179.1
30	−	17,403	18,350	Tail family protein	99%	0	98.41%	YP_009150258.1
31	−	18,366	21,131	Tape measure protein	99%	0	97.94%	QHB36803.1
32	−	21,139	21,426	Hypothetical protein	98%	3.00*E*-63	98.95%	YP_009147237.1
33	−	21,525	21,821	Hypothetical protein	98%	6.00*E*-52	96.94%	WP_136662160.1
34	−	21,849	22,493	Major tail protein	86%	5.00*E*-135	98.45%	YP_009149527.1
35	−	22,533	22,904	Hypothetical protein	99%	2.00*E*-77	94.31%	YP_009150015.1
36	−	22,901	23,191	Hypothetical protein	98%	7.00*E*-43	94.79%	YP_009151527.1
37	−	23,198	23,545	Hypothetical protein	99%	7.00*E*-78	98.26%	YP_009148279.1
38	−	23,547	24,008	Hypothetical protein	99%	8.00*E*-94	96.08%	YP_009146900.1
39	−	24,052	24,999	Capsid protein	90%	0	98.99%	YP_008531596.1
40	−	25,006	25,557	Scaffold protein	98%	7.00*E*-119	96.17%	ASJ79936.1
41	−	25,473	25,661	Hypothetical protein	98%	1.00*E*-25	77.42%	DAU50426.1
42	−	25,662	26,417	MuF-like minor capsid protein	99%	1.00*E*-164	96.02%	QHB36793.1
43	−	26,421	27,746	Portal protein	99%	0	98.87%	ASJ79938.1
44	−	27,743	29,254	Terminase large subunit	96%	0	97.54%	YP_009159829.1
45	−	29,259	29,507	Terminase small subunit	98%	5.00*E*-34	97.56%	QHB36790.1

ORF, open reading frame.

**Figure 6:**
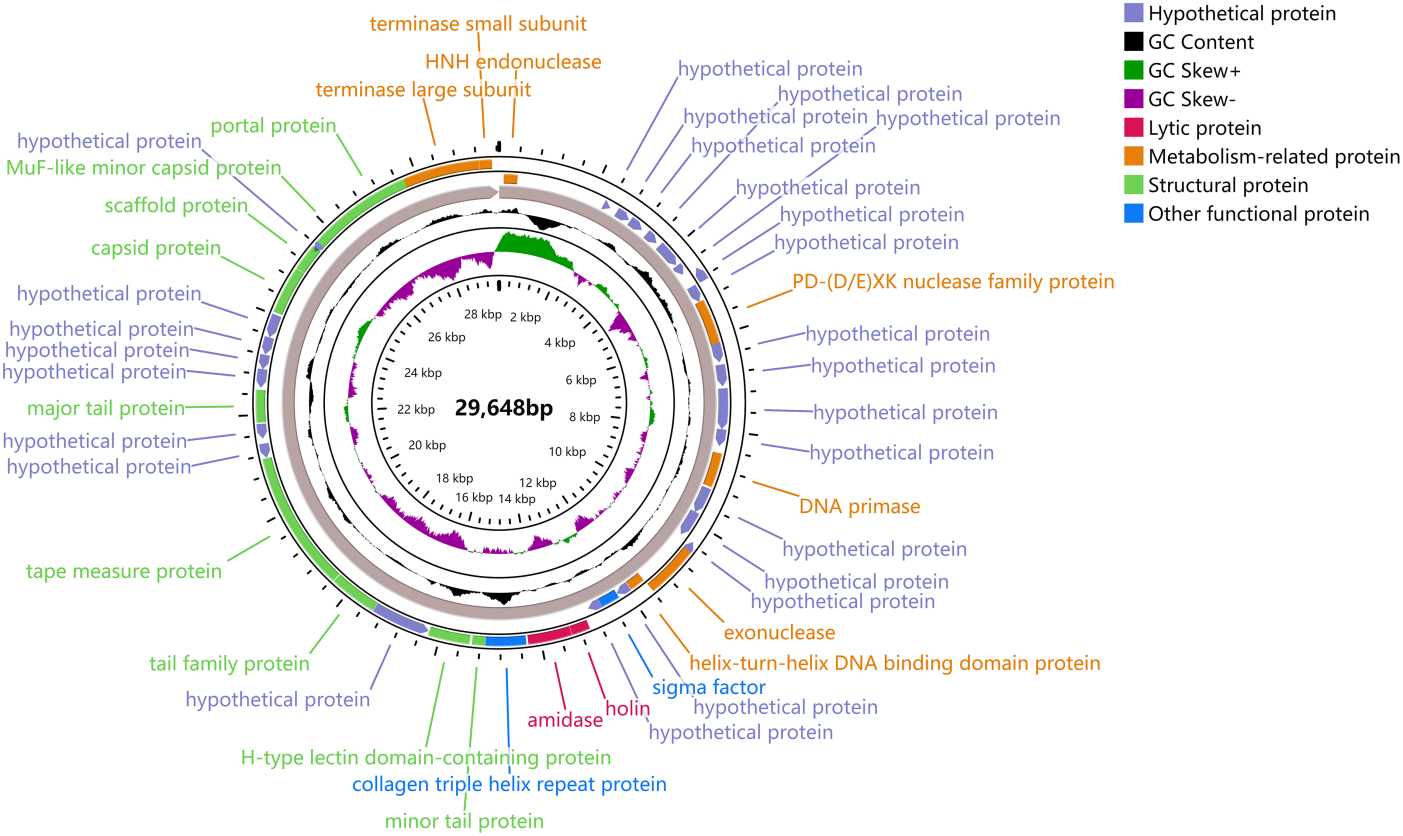
φPaP11-13 gene visualization. The middle number is the total length of the gene, and the circle diagram can be read from the inside out for G/C content, G/C ratio, transcription direction, and encoded protein. Different colors represent different functions. The outermost circles represent the 45 ORFs encoded by the genome, clockwise for the forward reading frame and counterclockwise for the reverse reading frame.

The predicted ORFs could be divided into four groups based on different functions: structural protein group, metabolism-related group, bacteria lysis group, and other functional groups. The structural protein contained minor tail protein (ORF34), H-type lectin domain-containing protein (ORF28), tail family protein (ORF30), tape measure protein (ORF31), major tail protein (ORF27), capsid protein (ORF39), scaffold protein (ORF40), MuF-like minor capsid protein (ORF42), and portal protein (ORF43). The metabolic-related group included HNH endonuclease (ORF1), P D-(D/E) X.K. nuclease family protein (ORF10), DNA Primase (ORF15), exonuclease (ORF19), helix-turn-helix DNA binding domain protein (ORF20), terminase large subunits (ORF44), and terminase small subunits (ORF45). The cleavage proteome included Amidase (ORF25) and Holin (ORF24). Other function groups include collagen triple helix repeat protein (ORF26) and sigma factor (ORF22). The annotation information of φPaP11-13 was uploaded to GenBank with the accession number: ON557706. The whole genome is visualized in Figure 6.

### Identification of phage proteins

**Figure 7:**
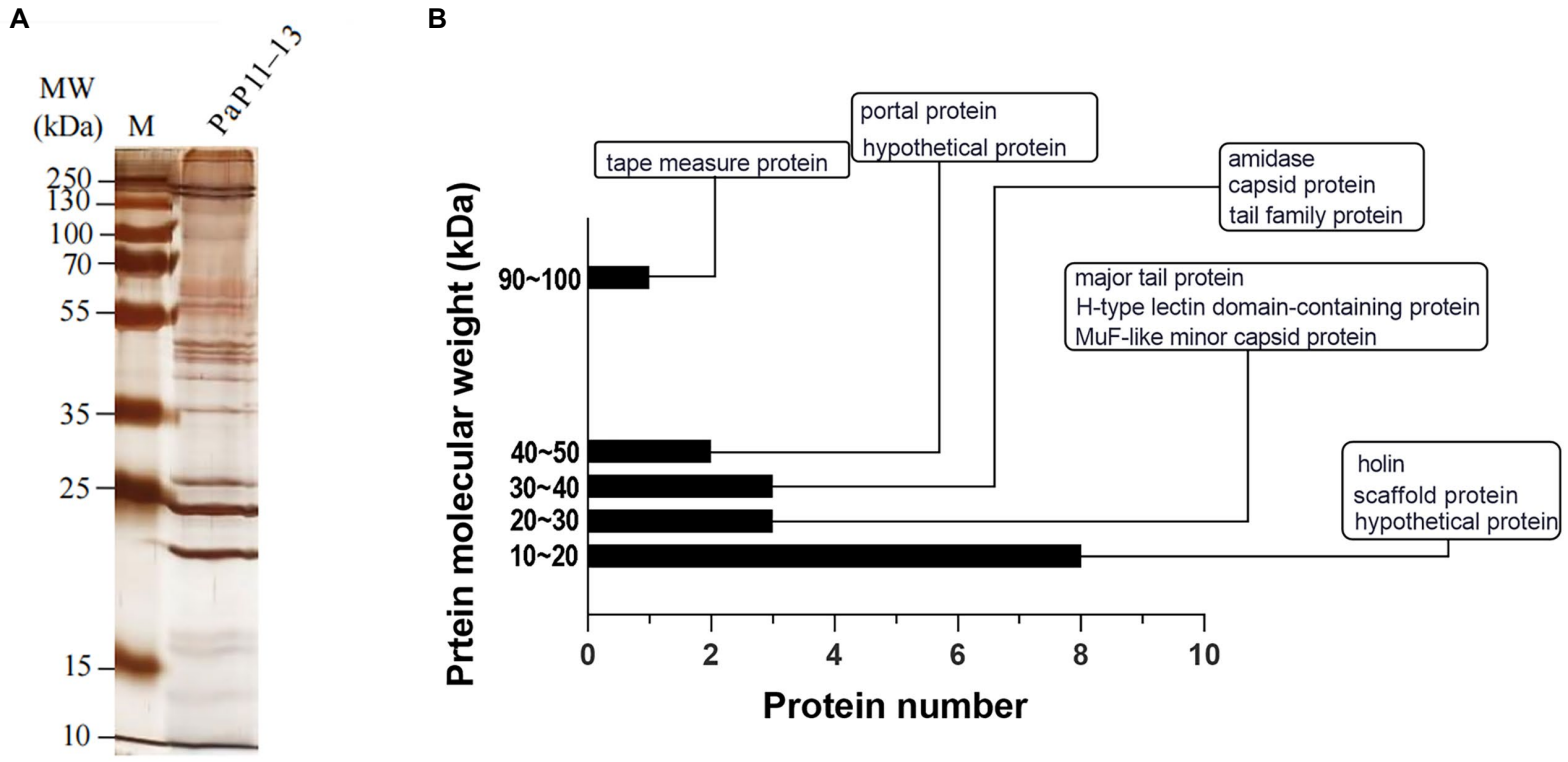
**(A)** SDS-PAGE, **(B)** Protein analysis of φPaP11-13. The molecular weight histogram of protein. The ordinate is the molecular weight of the protein, and the abscissa is the number of proteins. The indicator lines classify and label proteins.

SDS-PAGE was applied to verify the major proteins of φPaP11-13. The results showed clear protein bands and normal distribution (Figure 7A), indicating an undegraded phage protein preparation. Then all visible bands were cut and analyzed by LC–MS/MS system. 17 phage proteins with high intensity were identified (Table 5). As shown in Figure 7B, the bands were identified as the lysis-related proteins Holin (12.528 kDa, ORF24), Amidase (31.354 kDa, ORF25), and the tape measure protein (93.79 kDa, ORF31). Furthermore, the molecular weight between 20 and 30 kDa included scaffold protein (ORF40), major tail protein (ORF34), MuF-like minor capsid protein (ORF42), and H-type lectin domain-containing protein (ORF28); the molecular weight of 30–40 kDa included capsid protein (ORF39) and tail family protein (ORF30); molecular weight of 40–50 kDa included portal protein (ORF43).

**Table 5:** Protein mass spectrometry of φPaP11-13.

Protein description	MW(kDa)	Intensity
Holin	12.528	6,220,000
Amidase	31.354	69,798,000
H-type lectin domain-containing protein	28.751	977,880,000
Hypothetical protein	42.667	1,065,800,000
Tail family protein	34.939	1,138,100,000
Tape measure protein	93.79	304,070,000
Hypothetical protein	10.595	10,955,000
Major tail protein	23.06	15,828,000,000
Hypothetical protein	13.59	79,012,000
Hypothetical protein	13.109	4,946,200
Hypothetical protein	16.349	318,010,000
Capsid protein	32.709	20,596,000,000
Scaffold protein	19.838	74,398,000
MuF-like minor capsid protein	27.834	39,025,000
Portal protein	47.997	4,152,600,000
Hypothetical protein	14.965	26,995,000
Hypothetical protein	16.805	3,582,000

MW, molecular weight.

### Collinearity and phylogenetic analysis

**Figure 8:**
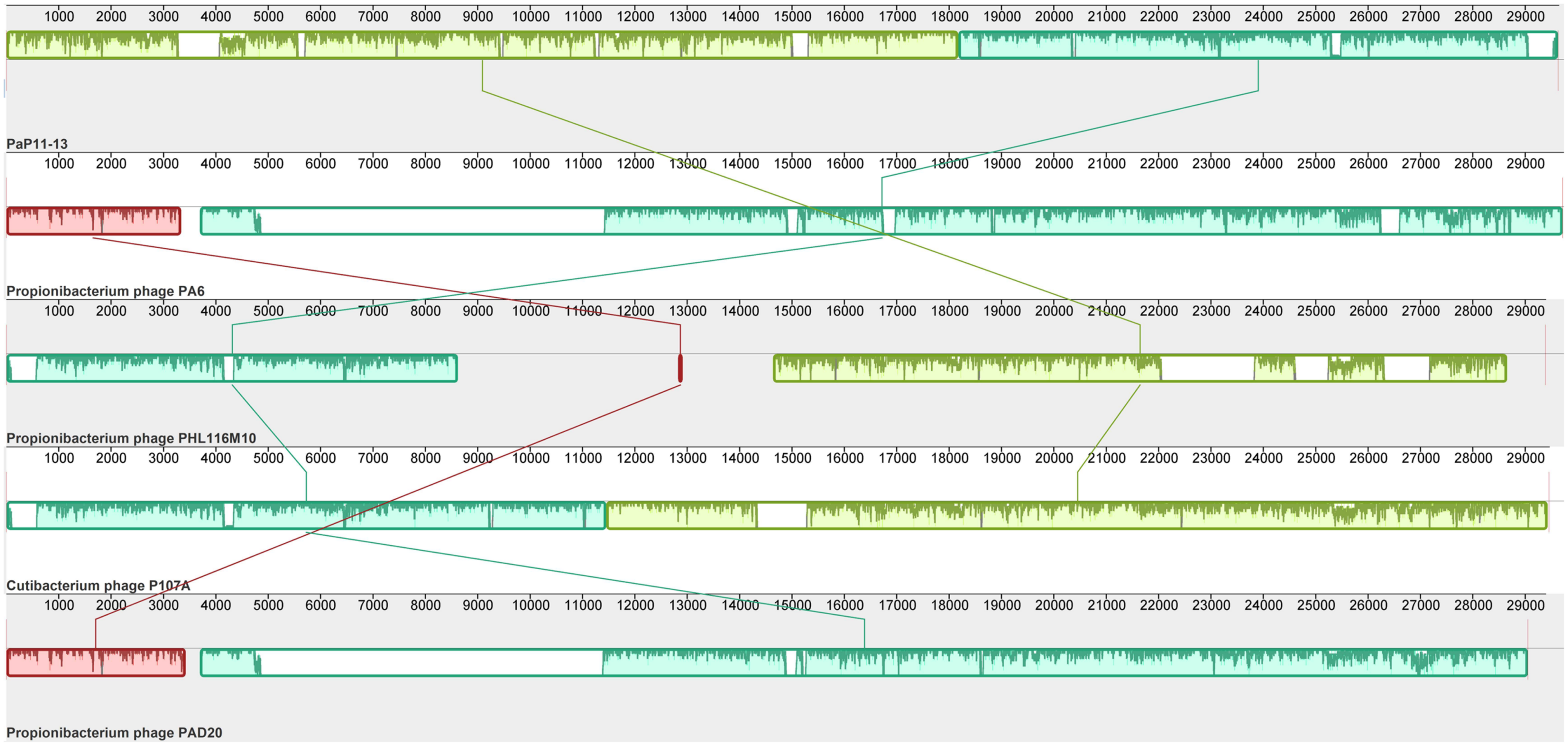
Collinearity analysis of φPaP11-13. From top to bottom are φPaP11-13, φPA6(98%), φPHL116M10(97%), φP107A(96%), φPAD20 (95%). The same color block represents the LCB. Blank regions inside and outside the LCB region represent regions of difference between genomes.

**Figure 9:**
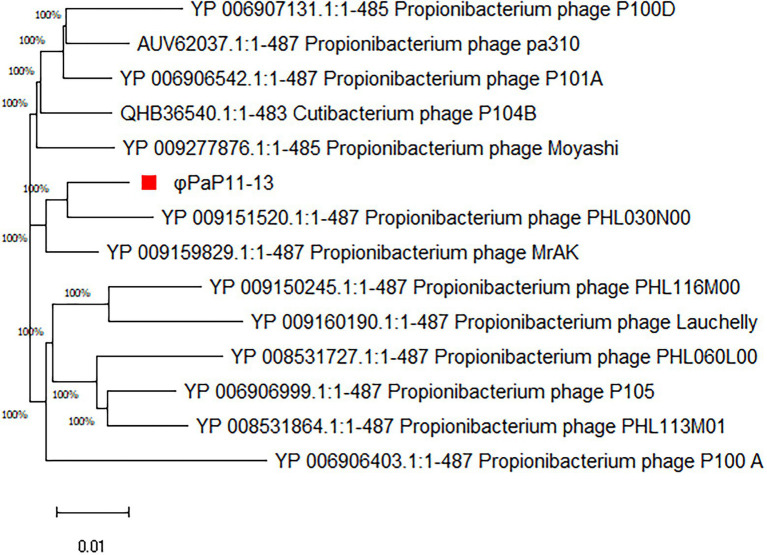
Phylogenetic analysis of φPaP11-13. The number in the phylogenetic tree is the phage accession number, and the number on the branch represents the reliability.

By comparing with the whole genome sequence in the NCBI database, the similarity between φPaP11-13 and known Sipoviridae family phages ranged from 41 to 98% (Supplementary Table S1). The two phages with the highest similarity to φPaP11-13 were φPA6 (dq4431235.1, 98%) and φct4Al2 (BK053742.1, 98%). However, the collinearity analysis revealed multiple local collinearity regions (LCB) between φPaP11-13 and these known phages. And these LCBs were rearranged and inverted. Even some genes within each LCB were distinct, with variants, rearrangements, and insertions (Figure 8). Collinearity analysis indicated that although φPaP11-13 was similar to these phages, there were significant internal differences (Orthologs and common conserved region sequence are listed in Supplementary Tables S2, S3). Phylogenetic trees were constructed based on the terminal large subunit protein because its amino acid sequence was highly conserved. As shown in Figure 9, φPaP11-13 and φPHL030N00 (KJ578760.1), a *P. acne* phage belonging to the Sipoviridae family, were on the same branch, with 100% reliability and short genetic distance, revealing the homology between φPaP11-13 and φPHL030N00. Collinearity and phylogenetic analysis confirmed *P. acne* phage φPaP11-13 as a member of the Sipoviridae family.

## Discussion

*P. acne* is not only a commensal bacterium that inhabits the sebaceous follicle but is also one of the primary acne triggers (Dreno et al., 2017). Prevention or removal of the *P. acne* infection can reduce the incidence and inflammation of severe acne vulgaris (Oge et al., 2019). The first-line treatment for severe acne vulgaris is still systematic antibiotic therapy (Hauk, 2017). But its side effects and increasing antibiotic resistance bring troubles. Antibiotic-resistant *P. acne*s-induced acne vulgaris has multiplied from 20% in the 20th century to 64% in the 21st century (Toyne et al., 2012) in Australia. Even in this study, we found 17 AMR genes in clinical strain, Pacne11-13, multi-drug resistant to almost all first-line antibiotics. The harsh situation brings trouble to clinical practice. Thus, it is urgent to explore new treatments for severe acne vulgaris caused by antibiotic-resistant *P. acne*. The discovery of *P. acnes* phage has a long history. In 1964, *P. acnes* phages were first identified (Brzin, 1964), and Zierdt et al. (1968) isolated phage 174 from *Corynebacterium acnes* strain and found that 88% of the *P. acnes* strain was sensitive to this phage. In 1974, they studied the biological characteristics of nine *P. acne* phages and found they were all with long, curved nonretractile tails and had good resistance to temperature and pH (Zierdt, 1974). In 2007, Farrar first reported the genome sequence of *P. acne* phage PA6 and demonstrated it without the virulence gene (Farrar et al., 2007). Phages are new alternatives to treating antibiotic-resistant bacteria (Ding et al., 2022). In this study, we isolated a lytic phage φPaP11-13 against a clinical multi-drug resistant *P. acne* strain Pacne11-13 with a typical Siphovirus morphology, icosahedral structure, an untraceable flexible tail, and a polyhedral head.

The one-step growth curve may provide intrinsic working rules of a target phage. However, there were few reports on one-step growth curves for *P. acne* phages but contradictory descriptions (Marinelli et al., 2012; Brüggemann and Lood, 2013). We managed to curve the one-step growth curve of φPaP11-13 after repeated experiments. *Propionibacterium* phages seem to have a more extended incubation time and a less rapid lysis period, which might be addressed to the host growth speed (Nakase et al., 2018).

From the application perspective, not only are particular phage screening and animal experiments increasing, but the corresponding clinical applications for treating diseases caused by drug-resistant bacteria are also increasing. In 2018, America reported a case of successful treatment of multidrug-resistant *Acinetobacter baumannii* infection with phages; In 2022, France reported the treatment of three patients with recurrent Staphylococcus aureus prosthesis knee infection (PKI), in which phage showed good efficacy (LaVergne et al., 2018; Ferry et al., 2022). Recently, a phase I clinical trial on topical applied *P. acne* phages treating antibiotic-resistant acne vulgaris successfully in the United States (Golembo et al., 2022). Besides, our team performed animal experiments demonstrating that phage therapy was as effective as antibiotic therapy. *P. acne* phages were also found to affect immunomodulators when evaluating the severity of inflammation of acne rats (data not shown). To understand the feasibility of phage therapy, it is necessary to comprehend phage’s biological characteristics. According to their characteristics, different administration methods, such as oral administration, injection, and application, are selected to reduce the influence on bacteriophage activity. Additionally, φPaP11-13 is functionally stable over a wide range of pH values and temperatures, potentially suitable in the human physiological environment. And the inactivation of φPaP11-13 under ultraviolet irradiation suggests a further application with sunscreen or at night to ensure the therapeutic effect on severe acne vulgaris. The stability of bacteriophages are mainly determined by their morphology and structure. Most *P. acne* phages reported belonged to the Siphoviridae family, sharing a similar morphology, with long tails and icosahedral heads, and thus performed the same stability (Zierdt, 1974; Marinelli et al., 2012). Due to the diversity and specificity between phages and their hosts, φPaP11-13 remains a potential candidate in our local country when the genomic background is thoroughly understood.

The genome of φPaP11-13 is linear dsDNA with a length of 29,648 bp, which is consistent with most phages of the Siphoviridae family. Although the similarity between the φPaP11-13 genome and known Sipoviridae family viruses ranged from 41 to 98%, our collinearity analysis suggests φPaP11-13 as a novel *P. acne* phage.

The high mutation rate of phages makes identifying gene function difficult (Gordillo Altamirano and Barr, 2019). Among the 45 predicted ORFs, only 20 ORFs have been annotated with their specific functions. The annotation suggests φPaP11-13 has two main lysis-related proteins: Holin (ORF25)and Amidase (ORF26), and their gene sequences are 99% similar to the known *P. acne* phages. They perform the lysis of host bacteria, which could produce synergistic effects during the lysis process (Fernandes and São-José, 2016) and even serve as independent antibiotics (Ghosh et al., 2019). Non-coding RNA genes and virulence factors were not found, making it safer for further applications. However, more transcriptomic analysis between the phage and host would help enrich an understanding and provide the basis for further research (Yang et al., 2019c).

## Conclusion

φPaP11-13 is a member of the Sipoviridae family, with good stability, strong lysis ability, and without virulence. It was identified as a new *P. acne* phage by biological characterization and genome analysis. Its discovery enriches the phage library of *P. acne* and provides the basis for the clinical application of phage therapy for antibiotics-resistant *P. acne*-induced severe acne vulgaris.

## Data availability statement

The datasets presented in this study can be found in online repositories. The names of the repository/repositories and accession number(s) can be found at: ncbi.nlm.nih.gov/nuccore/ON557706.

## Ethics statement

This is an original article on bacteriophages and bacteria. For this type of study, the requirement for ethics approval is waived by the Medical Ethics Committee of the Second Affiliated Hospital (Xinqiao Hospital) of Army Medical University, PLA.

## Author contributions

DL, YZ, and ZY conceived and designed the experiments. DL performed the experiments. JZ, RL, KC, YL, YS, and XS analyzed the data. DL and ZY wrote the paper. All authors contributed to the article and approved the submitted version.

## Funding

This work was supported by the National Natural Science Foundation of China (grant nos. 82002051), the Natural Science Foundation of Chongqing CSTC (cstc2021jcyj-msxmX0655), and the Doctor Through Line Project of Chongqing CSTB (CSTB2022BSXM-JCX0019).

## Conflict of interest

The authors declare that the research was conducted in the absence of any commercial or financial relationships that could be construed as a potential conflict of interest.

## Publisher’s note

All claims expressed in this article are solely those of the authors and do not necessarily represent those of their affiliated organizations, or those of the publisher, the editors and the reviewers. Any product that may be evaluated in this article, or claim that may be made by its manufacturer, is not guaranteed or endorsed by the publisher.

## Supplementary material

The Supplementary material for this article can be found online at: https://www.frontiersin.org/articles/10.3389/fmicb.2022.1065386/full#supplementary-material

Supplementary Figure S1 The grown curve of Pacne11-13.

## Footnotes

^1^ http://www.ncbi.nlm.nih.gov/

^2^ http://www.bioinformatics.org/cgview/gallery.html
