# Corrigendum: Whole-genome sequencing, annotation, and biological characterization of a novel Siphoviridae phage against multi-drug resistant *Propionibacterium acne*

**DOI:** 10.3389/fmicb.2024.1390825

**Published:** 2024-03-08

**Authors:** Danxi Liao, Jian Zhang, Ruolan Liu, Kui Chen, Yuanyuan Liu, Yuming Shao, Xi Shi, Yiming Zhang, Zichen Yang

**Affiliations:** ^1^Department of Plastic and Cosmetic Surgery, Xinqiao Hospital, The Second Affiliated Hospital, Army Medical University (The Third Military Medical University), Chongqing, China; ^2^Department of Clinical Laboratory, Xinqiao Hospital, The Second Affiliated Hospital, Army Medical University (The Third Military Medical University), Chongqing, China; ^3^Cadet Brigade 4, College of Basic Medicine, Army Medical University (The Third Military Medical University), Chongqing, China; ^4^Department of Microbiology, College of Basic Medicine, Army Medical University (The Third Military Medical University), Chongqing, China

**Keywords:** *Propionibacterium acne*, bacteriophage, severe acne vulgaris, antibiotics-resistant, phage therapy

In the published article, there was an error in the Funding statement. The funding statement “the Foundation of Open Projects from State Key Laboratory of Trauma, Burns and Combined Injury (No. SKLKF201918), the Novel Clinical Technology Category of Army Medical University (XJSZR1206), and the Talent Training Project of Army Medical University (2019R031)” was incorrect. The correct Funding statement appears below.

