# Supplementary figures and images for: Whole-genome sequencing, annotation, and biological characterization of a novel Siphoviridae phage against multi-drug resistant Propionibacterium acne
Source: Front Microbiol. 2023 Jan 4;13:1065386. doi: 10.3389/fmicb.2022.1065386 (PMC9846536; doi:10.3389/fmicb.2022.1065386)

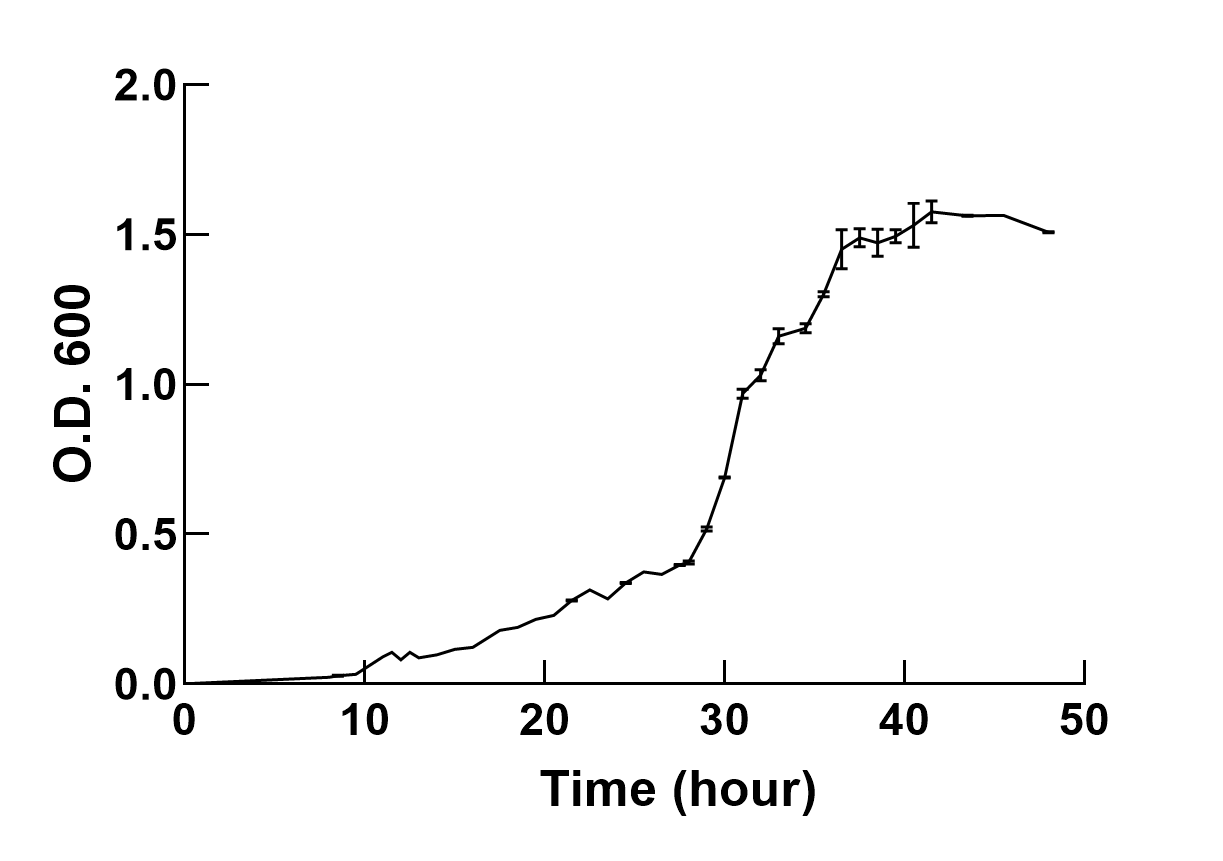

Supplement: Supplementary Figure S1 — The grown curve of Pacne11-13. [file Image_1.TIF]
