# Supplementary material for: Whole-genome sequencing, annotation, and biological characterization of a novel Siphoviridae phage against multi-drug resistant Propionibacterium acne
Source: Front Microbiol. 2023 Jan 4;13:1065386. doi: 10.3389/fmicb.2022.1065386 (PMC9846536; doi:10.3389/fmicb.2022.1065386)
Supplement: Supplementary file 2 [file Table_6.DOCX]

**Supplementary Table S1.** **The similarity between φPaP11-13 and known Sipoviridae family phages from NCBI.**

| **Description** | **Query Cover** | **E value** | **Accession** |
| --- | --- | --- | --- |
| TPA: Siphoviridae sp. isolate ct4Al2 | 98% | 0 | BK053742.1 |
| Propionibacterium phage PA6 | 98% | 0 | DQ431235.1 |
| Propionibacterium phage PHL116M10 | 97% | 0 | KJ578777.1 |
| Propionibacterium phage PHL116M00 | 97% | 0 | KJ578776.1 |
| Propionibacterium phage PHL095N00 | 97% | 0 | KJ578774.1 |
| Propionibacterium phage PHL071N05 | 97% | 0 | JX570710.1 |
| Propionibacterium phage P14.4 | 97% | 0 | NC_018839.1 |
| Propionibacterium phage Ouroboros | 97% | 0 | NC_027630.1 |
| Propionibacterium phage pa27 | 97% | 0 | MG820634.1 |
| Propionibacterium phage PHL308M00 | 97% | 0 | NC_027376.1 |
| Propionibacterium phage TCUCAP1 | 97% | 0 | MW505928.1 |
| Propionibacterium phage PHL150M00 | 97% | 0 | KJ578782.1 |
| TPA: Siphoviridae sp. isolate ctm362 | 97% | 0 | BK044867.1 |
| Cutibacterium phage P108C | 97% | 0 | MN813683.1 |
| Propionibacterium phage Leviosa | 97% | 0 | MF919515.1 |
| Propionibacterium phage pa6919-4 | 97% | 0 | MG820638.1 |
| Propionibacterium phage PHL085N00 | 97% | 0 | KJ578772.1 |
| Propionibacterium phage pa9-6919-4 | 97% | 0 | MG820639.1 |
| Propionibacterium phage PHL037M02 | 97% | 0 | NC_022339.1 |
| Propionibacterium phage PHL115M02 | 97% | 0 | JX570708.1 |
| Propionibacterium phage PHL085M01 | 97% | 0 | JX570707.1 |
| TPA: Siphoviridae sp. isolate ct0fI1 | 97% | 0 | BK054346.1 |
| Propionibacterium phage pa28 | 97% | 0 | MG820642.1 |
| Propionibacterium phage Pirate | 97% | 0 | NC_027623.2 |
| TPA: Siphoviridae sp. isolate ctulL1 | 97% | 0 | BK046057.1 |
| TPA: Siphoviridae sp. isolate ctlEX2 | 97% | 0 | BK048405.1 |
| Cutibacterium phage P107A | 96% | 0 | MN813679.1 |
| Propionibacterium phage PacnesP1 | 96% | 0 | KY926792.1 |
| Propionibacterium phage Solid | 96% | 0 | NC_027627.1 |
| Propionibacterium phage PHL171M01 | 96% | 0 | KJ578787.1 |
| Propionibacterium phage Lauchelly | 96% | 0 | NC_027628.1 |
| Propionibacterium phage pa310 | 96% | 0 | MG820632.1 |
| Propionibacterium phage pa59 | 96% | 0 | MG820633.1 |
| Cutibacterium phage P104B | 96% | 0 | MN813675.1 |
| Propionibacterium phage P1.1 | 96% | 0 | NC_018842.1 |
| TPA: Siphoviridae sp. ctkV91 | 96% | 0 | BK032807.1 |
| Propionibacterium phage PHL070N00 | 96% | 0 | KJ578767.1 |
| Propionibacterium phage P100D | 96% | 0 | NC_018852.1 |
| Propionibacterium phage PHL112N00 | 96% | 0 | JX570714.1 |
| Propionibacterium phage BruceLethal | 96% | 0 | NC_031084.1 |
| Propionibacterium phage pa615 | 96% | 0 | MG820641.1 |
| Propionibacterium phage pa15 | 96% | 0 | MG820640.1 |
| Propionibacterium phage PHL060L00 | 96% | 0 | JX570705.1 |
| Propionibacterium phage PAD20 | 95% | 0 | FJ706171.1 |
| Propionibacterium phage Attacne | 95% | 0 | NC_027629.1 |
| Propionibacterium phage pa29399-1-D_2 | 95% | 0 | MG820636.1 |
| Propionibacterium phage PHL064M01 | 95% | 0 | KJ578763.1 |
| Propionibacterium phage PHL064M02 | 95% | 0 | KJ578764.1 |
| Propionibacterium phage PHL030N00 | 95% | 0 | KJ578760.1 |
| Propionibacterium phage PHL117M01 | 95% | 0 | NC_041956.1 |
| TPA: Siphoviridae sp. isolate ct0kU1 | 95% | 0 | BK051286.1 |
| Propionibacterium phage PHL092M00 | 95% | 0 | KJ578773.1 |
| Propionibacterium phage PHL163M00 | 95% | 0 | NC_027405.1 |
| Propionibacterium phage PHL117M00 | 95% | 0 | NC_027403.1 |
| Propionibacterium phage PHL194M00 | 95% | 0 | NC_027392.1 |
| Propionibacterium phage PHL179M00 | 95% | 0 | KJ578788.1 |
| Propionibacterium phage PHL141N00 | 95% | 0 | KJ578781.1 |
| Cutibacterium phage P107C | 95% | 0 | MN813677.1 |
| Propionibacterium phage ATCC29399B_C | 95% | 0 | NC_018851.1 |
| Propionibacterium phage PHL067M10 | 95% | 0 | JX570709.1 |
| Propionibacterium phage PHL067M01 | 95% | 0 | NC_027380.1 |
| Propionibacterium phage PHL067M09 | 95% | 0 | KJ578766.1 |
| Propionibacterium phage PHL111M01 | 95% | 0 | JX570702.1 |
| Propionibacterium phage pa29399-1-D_1 | 95% | 0 | MG820635.1 |
| Propionibacterium phage Procrass1 | 95% | 0 | NC_027626.1 |
| Propionibacterium phage P100_A | 95% | 0 | NC_018838.1 |
| Propionibacterium phage PHL073M02 | 95% | 0 | JX570703.1 |
| Propionibacterium phage PHL151N00 | 95% | 0 | NC_041957.1 |
| Propionibacterium phage PHL151M00 | 95% | 0 | NC_027347.1 |
| Propionibacterium phage PHL066M04 | 95% | 0 | JX570711.1 |
| Propionibacterium phage PHL010M04 | 95% | 0 | JX570704.1 |
| Propionibacterium phage P9.1 | 95% | 0 | NC_018834.1 |
| Propionibacterium phage PHL114N00 | 95% | 0 | KJ578775.1 |
| Propionibacterium phage PHL114L00 | 95% | 0 | JX570712.1 |
| Propionibacterium phage Moyashi | 95% | 0 | NC_031003.1 |
| Propionibacterium phage ATCC29399B_T | 95% | 0 | NC_018847.1 |
| Propionibacterium phage pa63 | 95% | 0 | MG820637.1 |
| Propionibacterium phage Stormborn | 95% | 0 | NC_027622.1 |
| Propionibacterium phage Supernova | 95% | 0 | MF919533.1 |
| Propionibacterium phage LilBandit | 95% | 0 | MF919516.1 |
| Propionibacterium phage Enoki | 95% | 0 | NC_031119.1 |
| Propionibacterium phage Aquarius | 95% | 0 | MF919491.1 |
| Propionibacterium phage P104A | 94% | 0 | NC_018845.1 |
| Propionibacterium phage PAS50 | 94% | 0 | FJ706172.1 |
| Propionibacterium phage PHL113M01 | 94% | 0 | JX570713.1 |
| Propionibacterium phage QueenBey | 93% | 0 | NC_031005.2 |
| Propionibacterium phage MEAK | 93% | 0 | MF919522.1 |
| Propionibacterium phage PHL152M00 | 93% | 0 | KJ578785.1 |
| Propionibacterium phage Wizzo | 93% | 0 | NC_027621.1 |
| Propionibacterium phage pa3-SS3 | 91% | 0 | MG820645.1 |
| Propionibacterium phage pa33 | 91% | 0 | MG820644.1 |
| Propionibacterium phage PHL199M00 | 88% | 0 | KJ578790.1 |
| TPA: Siphoviridae sp. isolate ct9aH1 | 78% | 0 | BK029815.1 |
| TPA: Siphoviridae sp. isolate ctufG1 | 77% | 0 | BK041711.1 |
| TPA: Siphoviridae sp. isolate ctptf1 | 73% | 0 | BK038489.1 |
| TPA: Siphoviridae sp. isolate ctHDD1 | 51% | 0 | BK035457.1 |
| TPA: Siphoviridae sp. isolate ctahB1 | 51% | 0 | BK031062.1 |
| TPA: Siphoviridae sp. isolate ctTIN2 | 46% | 0 | BK051042.1 |
| TPA: Siphoviridae sp. isolate ctMw21 | 45% | 0 | BK041953.1 |
| TPA: Siphoviridae sp. isolate ctXNH1 | 41% | 0 | BK038603.1 |
