# Supplementary material for: Whole-genome sequencing, annotation, and biological characterization of a novel Siphoviridae phage against multi-drug resistant Propionibacterium acne
Source: Front Microbiol. 2023 Jan 4;13:1065386. doi: 10.3389/fmicb.2022.1065386 (PMC9846536; doi:10.3389/fmicb.2022.1065386)
Supplement: Supplementary file 3 [file Table_7.DOCX]

**Supplementary Table S2. Orthologs between φPaP11-13 and known Sipoviridae family phages.**

| **φPaP11-13** | | | **φPA6** | | | **φPHL116M10** | | | **φP107A** | | | **φPAD20** | | | |
| --- | --- | --- | --- | --- | --- | --- | --- | --- | --- | --- | --- | --- | --- | --- | --- |
| **Start** | **End** | **Length** | **Start** | **End** | **Length** | **Start** | **End** | **Length** | **Start** | **End** | **Length** | **Start** | **End** | **Length** |  |
| 97 | 386 | 289 | 29388 | 29690 | 302 | 29043 | 29345 | 302 | 29101 | 29403 | 302 | 28723 | 29025 | 302 |  |
| 2288 | 2449 | 161 | 27430 | 27612 | 182 | 27033 | 27176 | 143 |  |  |  |  |  |  |  |
| 2606 | 2914 | 308 | 27007 | 27273 | 266 | 26572 | 26880 | 308 | 26614 | 26922 | 308 | 26387 | 26653 | 266 |  |
| 2946 | 3257 | 311 | 26622 | 26933 | 311 | 26225 | 26539 | 314 | 26263 | 26574 | 311 | 26000 | 26311 | 311 |  |
| 3386 | 3670 | 284 | 26206 | 26493 | 287 |  |  |  |  |  |  |  |  |  |  |
| 3754 | 4290 | 536 | 25581 | 26120 | 539 | 25571 | 26104 | 533 | 25607 | 26140 | 533 | 25343 | 25876 | 533 |  |
| 4315 | 4542 | 227 | 25331 | 25558 | 227 | 25321 | 25548 | 227 | 25363 | 25590 | 227 | 25091 | 25318 | 227 |  |
| 4851 | 5210 | 359 | 24666 | 25025 | 359 | 24656 | 25015 | 359 | 24707 | 25060 | 353 | 24432 | 24785 | 353 |  |
| 5210 | 6151 | 941 | 23719 | 24666 | 947 | 23709 | 24656 | 947 | 23766 | 24707 | 941 | 23503 | 24432 | 929 |  |
| 6148 | 6558 | 410 | 23312 | 23722 | 410 | 23302 | 23712 | 410 | 23359 | 23769 | 410 | 23078 | 23488 | 410 |  |
| 6610 | 7068 | 458 | 22791 | 23261 | 470 | 22797 | 23261 | 464 | 22841 | 23308 | 467 | 22572 | 23036 | 464 |  |
| 7111 | 7974 | 863 | 21796 | 22749 | 953 | 21803 | 22756 | 953 | 21941 | 22804 | 863 | 21666 | 22529 | 863 |  |
| 7971 | 8330 | 359 | 21530 | 21886 | 356 |  |  |  | 21585 | 21941 | 356 | 21310 | 21666 | 356 |  |
| 8474 | 9199 | 725 | 20661 | 21332 | 671 | 20668 | 21339 | 671 | 20716 | 21387 | 671 | 20441 | 21112 | 671 |  |
| 9244 | 9810 | 566 | 20053 | 20616 | 563 | 20050 | 20616 | 566 | 20106 | 20672 | 566 | 19833 | 20396 | 563 |  |
| 9807 | 10367 | 560 | 19513 | 19953 | 440 | 19496 | 20053 | 557 | 19552 | 20109 | 557 | 19273 | 19836 | 563 |  |
| 10351 | 10545 | 194 | 19312 | 19506 | 194 | 19318 | 19512 | 194 | 19374 | 19568 | 194 | 19095 | 19289 | 194 |  |
| 10542 | 11588 | 1046 | 18269 | 19315 | 1046 | 18281 | 19321 | 1040 | 18331 | 19377 | 1046 | 18052 | 19098 | 1046 |  |
| 11598 | 11918 | 320 | 17938 | 18258 | 320 | 17951 | 18271 | 320 | 18001 | 18321 | 320 | 17721 | 18041 | 320 |  |
| 11930 | 12217 | 287 | 17641 | 17916 | 275 | 17664 | 17939 | 275 | 17702 | 17986 | 284 | 17425 | 17706 | 281 |  |
| 12221 | 12619 | 398 | 17245 | 17637 | 392 | 17262 | 17660 | 398 | 17297 | 17698 | 401 | 17033 | 17428 | 395 |  |
| 12624 | 12899 | 275 | 16984 | 17217 | 233 |  |  |  |  |  |  |  |  |  |  |
| 13022 | 13384 | 362 | 16249 | 16650 | 401 | 16279 | 16677 | 398 | 16299 | 16697 | 398 | 16265 | 16669 | 404 |  |
| 13391 | 14254 | 863 | 15376 | 16236 | 860 | 15415 | 16272 | 857 | 15432 | 16286 | 854 | 15389 | 16252 | 863 |  |
| 14294 | 15091 | 797 | 14630 | 15319 | 689 | 14558 | 15364 | 806 | 14571 | 15377 | 806 | 14599 | 15339 | 740 |  |
| 15094 | 15357 | 263 | 14331 | 14570 | 239 | 14289 | 14555 | 266 | 14296 | 14559 | 263 | 14300 | 14539 | 239 |  |
| 15403 | 16221 | 818 | 13444 | 14262 | 818 | 13417 | 14247 | 830 | 13430 | 14248 | 818 | 13413 | 14231 | 818 |  |
| 16238 | 17395 | 1157 | 12270 | 13427 | 1157 | 12255 | 13412 | 1157 | 12255 | 13412 | 1157 | 12238 | 13395 | 1157 |  |
| 17403 | 18350 | 947 | 11315 | 12262 | 947 | 11285 | 12247 | 962 | 11301 | 12248 | 947 | 11289 | 12230 | 941 |  |
| 18366 | 21131 | 2765 | 8532 | 11297 | 2765 | 8519 | 11284 | 2765 | 8520 | 11285 | 2765 | 8508 | 11273 | 2765 |  |
| 21139 | 21426 | 287 | 8237 | 8524 | 287 | 8224 | 8511 | 287 |  |  |  | 8213 | 8500 | 287 |  |
| 21525 | 21821 | 296 | 7842 | 8138 | 296 | 7829 | 8125 | 296 | 7830 | 8126 | 296 | 7821 | 8114 | 293 |  |
| 21849 | 22493 | 644 | 7172 | 7813 | 641 | 7166 | 7801 | 635 | 7168 | 7800 | 632 | 7164 | 7793 | 629 |  |
| 22533 | 22904 | 371 | 6766 | 7113 | 347 | 6746 | 7117 | 371 | 6743 | 7114 | 371 | 6740 | 7111 | 371 |  |
| 22901 | 23191 | 290 | 6455 | 6745 | 290 | 6459 | 6749 | 290 | 6456 | 6746 | 290 | 6453 | 6743 | 290 |  |
| 23198 | 23545 | 347 | 6101 | 6448 | 347 | 6104 | 6451 | 347 | 6102 | 6449 | 347 | 6099 | 6446 | 347 |  |
| 23547 | 24008 | 461 | 5638 | 6099 | 461 | 5623 | 6102 | 479 | 5639 | 6100 | 461 | 5636 | 6097 | 461 |  |

These are the phage genes involved in collinearity analysis by Mauve 20150226.

From left to right are φPaP11-13, φPA6（98%）, φPHL116M10（97%）, φP107A(96%), φPAD20 (95%).
