# Supplementary material for: Whole-genome sequencing, annotation, and biological characterization of a novel Siphoviridae phage against multi-drug resistant Propionibacterium acne
Source: Front Microbiol. 2023 Jan 4;13:1065386. doi: 10.3389/fmicb.2022.1065386 (PMC9846536; doi:10.3389/fmicb.2022.1065386)
Supplement: Supplementary file 4 [file Table_8.DOCX]

**Supplementary Table S3. Common conserved region sequence between φPaP11-13 and known Sipoviridae family phages.**

| **φPaP11-13** | | | **φPA6** | | | **φPHL116M10** | | | **φP107A** | | | **φPAD20** | | |
| --- | --- | --- | --- | --- | --- | --- | --- | --- | --- | --- | --- | --- | --- | --- |
| **Start** | **End** | **Length** | **Start** | **End** | **Length** | **Start** | **End** | **Length** | **Start** | **End** | **Length** | **Start** | **End** | **Length** |
| 17859 | 18033 | 174 | - | - | - | 11791 | 11618 | 173 | 11792 | 11619 | 173 | - | - | - |
| 2135 | 2188 | 53 | - | - | - | 27361 | 27307 | 54 | 27400 | 27348 | 52 | - | - | - |
| 15511 | 15531 | 20 | - | - | - | 14139 | 14120 | 19 | 14140 | 14121 | 19 | - | - | - |
| 3264 | 3639 | 375 | 26615 | 26237 | 378 | - | - | - | - | - | - | - | - | - |
| 2189 | 2303 | 114 | 27676 | 27563 | 113 | 27306 | 27191 | 115 | 27347 | 27233 | 114 | - | - | - |
| 2304 | 2332 | 28 | - | - | - | 27191 | 27162 | 29 | 27233 | 27204 | 29 | - | - | - |
| 2333 | 2341 | 8 | 27558 | 27550 | 8 | 27161 | 27153 | 8 | 27203 | 27195 | 8 | - | - | - |
| 12877 | 12887 | 10 | 16987 | 16974 | 13 | 17025 | 17012 | 13 | 17038 | 17025 | 13 | - | - | - |
| 1324 | 1387 | 63 | 28529 | 28464 | 65 | 28173 | 28103 | 70 | - | - | - | - | - | - |
| 999 | 1018 | 19 | 28754 | 28720 | 34 | - | - | - | 28463 | 28438 | 25 | - | - | - |
| 1118 | 1144 | 26 | - | - | - | - | - | - | 28339 | 28319 | 20 | - | - | - |
| 1234 | 1318 | 84 | - | - | - | 28263 | 28178 | 85 | 28238 | 28159 | 79 | - | - | - |
| 1319 | 1323 | 4 | 28533 | 28529 | 4 | 28177 | 28173 | 4 | 28158 | 28154 | 4 | - | - | - |
| 2131 | 2134 | 3 | 27735 | 27731 | 4 | 27365 | 27361 | 4 | 27404 | 27400 | 4 | - | - | - |
| 27802 | 29637 | 1835 | 1830 | 1 | 1829 | 1830 | 1 | 1829 | 1838 | 1 | 1837 | 1830 | 1 | 1829 |
| 27782 | 27801 | 19 | - | - | - | 1851 | 1831 | 20 | 1859 | 1839 | 20 | 1851 | 1831 | 20 |
| 22036 | 27781 | 5745 | 7617 | 1851 | 5766 | 7614 | 1851 | 5763 | 7613 | 1859 | 5754 | 7609 | 1851 | 5758 |
| 22022 | 22035 | 13 | 7632 | 7618 | 14 | - | - | - | 7628 | 7614 | 14 | 7624 | 7610 | 14 |
| 18308 | 22021 | 3713 | 11357 | 7632 | 3725 | 11342 | 7629 | 3713 | 11343 | 7628 | 3715 | 11331 | 7624 | 3707 |
| 18034 | 18289 | 255 | 11631 | 11376 | 255 | 11616 | 11361 | 255 | 11617 | 11362 | 255 | 11599 | 11350 | 249 |
| 15532 | 17858 | 2326 | 14133 | 11807 | 2326 | 14118 | 11792 | 2326 | 14119 | 11793 | 2326 | 14102 | 11775 | 2327 |
| 14614 | 15510 | 896 | 15050 | 14155 | 895 | 15035 | 14140 | 895 | 15039 | 14141 | 898 | 15019 | 14124 | 895 |
| 14592 | 14613 | 21 | - | - | - | 15057 | 15036 | 21 | 15061 | 15040 | 21 | 15041 | 15020 | 21 |
| 14411 | 14564 | 153 | 15262 | 15082 | 180 | 15247 | 15085 | 162 | 15260 | 15098 | 162 | 15222 | 15069 | 153 |
| 14352 | 14410 | 58 | - | - | - | 15307 | 15248 | 59 | 15320 | 15261 | 59 | 15282 | 15223 | 59 |
| 12907 | 14351 | 1444 | 16729 | 15266 | 1463 | 16755 | 15307 | 1448 | 16771 | 15320 | 1451 | 16745 | 15282 | 1463 |
| 12744 | 12876 | 132 | 17120 | 16988 | 132 | 17158 | 17026 | 132 | 17171 | 17039 | 132 | 16908 | 16776 | 132 |
| 12719 | 12743 | 24 | 17146 | 17121 | 25 | - | - | - | 17197 | 17172 | 25 | 16934 | 16909 | 25 |
| 11161 | 12718 | 1557 | 18696 | 17146 | 1550 | 18708 | 17163 | 1545 | 18758 | 17197 | 1561 | 18479 | 16934 | 1545 |
| 11136 | 11160 | 24 | 18721 | 18697 | 24 | - | - | - | 18783 | 18759 | 24 | 18504 | 18480 | 24 |
| 11132 | 11135 | 3 | 18726 | 18722 | 4 | - | - | - | - | - | - | 18509 | 18505 | 4 |
| 11115 | 11131 | 16 | 18743 | 18726 | 17 | 18755 | 18738 | 17 | - | - | - | 18526 | 18509 | 17 |
| 3682 | 11114 | 7432 | 26194 | 18743 | 7451 | 26176 | 18755 | 7421 | 26214 | 18805 | 7409 | 25950 | 18526 | 7424 |
| 3641 | 3681 | 40 | 26235 | 26195 | 40 | - | - | - | - | - | - | 25991 | 25951 | 40 |
| 2342 | 3261 | 919 | 27549 | 26618 | 931 | 27152 | 26221 | 931 | 27194 | 26259 | 935 | 26933 | 25996 | 937 |
| 1394 | 2130 | 736 | 28457 | 27735 | 722 | 28096 | 27365 | 731 | 28149 | 27404 | 745 | 27857 | 27148 | 709 |
| 1388 | 1393 | 5 | 28463 | 28458 | 5 | 28102 | 28097 | 5 | - | - | - | 27863 | 27858 | 5 |
| 1224 | 1233 | 9 | - | - | - | 28273 | 28263 | 10 | 28244 | 28238 | 6 | 27913 | 27903 | 10 |
| 1145 | 1223 | 78 | 28616 | 28538 | 78 | 28351 | 28273 | 78 | 28318 | 28244 | 74 | 27982 | 27913 | 69 |
| 1019 | 1117 | 98 | 28718 | 28620 | 98 | 28451 | 28353 | 98 | 28436 | 28339 | 97 | 28082 | 27984 | 98 |
| 560 | 997 | 437 | 29183 | 28756 | 427 | 28887 | 28454 | 433 | 28896 | 28464 | 432 | 28519 | 28085 | 434 |
| 529 | 559 | 30 | 29216 | 29184 | 32 | - | - | - | 28929 | 28897 | 32 | 28552 | 28520 | 32 |
| 401 | 528 | 127 | 29343 | 29216 | 127 | 29018 | 28892 | 126 | 29056 | 28929 | 127 | 28678 | 28552 | 126 |
| 377 | 400 | 23 | 29368 | 29344 | 24 | - | - | - | 29081 | 29057 | 24 | 28703 | 28679 | 24 |
| 3 | 376 | 373 | 29739 | 29368 | 371 | 29394 | 29023 | 371 | 29452 | 29081 | 371 | 29074 | 28703 | 371 |

These are the phage genes involved in collinearity analysis by Mauve 20150226.

From left to right are φPaP11-13, φPA6（98%）, φPHL116M10（97%）, φP107A(96%), φPAD20 (95%).
